# Frailty in Community-Dwelling Older People in Abu Dhabi, United Arab Emirates: A Cross-Sectional Study

**DOI:** 10.3389/fpubh.2015.00248

**Published:** 2015-11-03

**Authors:** Saleha Jaber Al-Kuwaiti, Faisal Aziz, Iain Blair

**Affiliations:** ^1^Zayed Military Hospital, Abu Dhabi, United Arab Emirates; ^2^Institute of Public Health, College of Medicine and Health Sciences, United Arab Emirates University, Al Ain, United Arab Emirates

**Keywords:** frailty, prevalence, United Arab Emirates, Abu Dhabi, Emirati, community dwelling

## Abstract

**Background:**

Frailty describes the aging-associated loss of physiological and psychological reserves, leading to an increased risk of adverse health outcomes. Many developed countries view frailty as a major priority for their health and social care systems. Less is known about frailty in less-developed countries. The purpose of this study was to determine the prevalence of frailty in a sample of community-dwelling older people in the United Arab Emirates (UAE).

**Methods:**

This was a cross-sectional study of community-dwelling Emirati adults aged 55 years and older (*n* = 160) in Abu Dhabi, UAE. Data were collected at interview by questionnaire and physical measurements. Frailty was defined according to the criteria of the Fried Frailty Index. The prevalence of frailty and its association with selected independent variables were assessed.

**Results:**

The overall prevalence of frailty (95% CI) was 47% (39–55). Higher levels of frailty were seen in older age groups, women, those who were non-married, those with recent hospital admission, those with comorbid conditions, those on more than five medications, and those with lower forced expiratory volume and mini-mental state examination score. After adjustment in a multiple logistic regression model, only age and gender were found to be independently associated with frailty.

**Conclusion:**

A high prevalence of frailty was found among older Emiratis. Given that frailty is associated with adverse health outcomes and can be a means of identifying opportunities for intervention in clinical practice and health policy, further attention and consideration within professional and public health policy circles are needed.

## Introduction

Not everyone can “age well,” persons with the same chronological age may vary according to health and functional status. Although there is no single accepted definition, “frailty” is used to describe the multidimensional syndrome in which there is a loss of physiological and psychological reserves leading to increased vulnerability to adverse health outcomes, such as falls, loss of mobility and independence, hospitalization, and death (1).

Frailty is important for both clinicians and health service managers because it identifies groups of people who need different types of intervention and higher levels of health and social care (2, 3).

It is generally accepted that the prevalence of frailty increases with age and is higher in women and those with chronic disease. However, estimates vary due to the different definitions of frailty that are in use.

In epidemiological studies, frailty is operationalized in two main ways. First, as a frailty phenotype such as the Fried Frailty Index (FFI) based on the presence of three or more of five components (unintentional weight loss, self-reported exhaustion, weakness, slow walking speed, and low physical activity) (4). Second, using a definition that includes psychosocial attributes such as the frailty index (FI), which is based on routine clinical assessment and lists deficits in selected areas such as mood, cognition, and continence (5, 6).

Many variations of these definitions can be found in the literature along with descriptions of the instruments that will measure them. Most definitions also allow for an intermediate pre-frailty state. The different definitions of frailty result in the reporting of a wide range of frailty prevalence between studies.

Population aging is occurring throughout the world and currently persons aged 60 or over account for 12% of the global population while those aged 80+ account for 1.7% (7). The corresponding percentages in a highly developed country such as Germany are 27.6 and 5.7%.

Faced with an aging population and an increasing number of frail elderly persons, many developed countries now view frailty as a major priority for their health and social care systems. This has resulted in a growth in frailty research including efforts to accurately estimate the prevalence of frailty using well-designed epidemiological surveys (8).

For example, a review that pooled the results of 21 studies found that among over 60,000 community-dwelling older persons, the average prevalence of frailty was 10.7% and a further 41.6% were pre-frail (9). Amongst the studies, which were conducted in Europe, US, Canada, Australia, and Taiwan, rates varied greatly from 4 to 60%. A physical definition of frailty, either the FFI or a modification, was the most commonly used definition of frailty, and in studies using this definition, average prevalence was 10% compared to 13.6% in studies using a broader definition. Among women, the prevalence of frailty and pre-frailty was 9.6 and 39%, respectively, while among men it was 5.2 and 37%. Frailty increased with age, the prevalence in those aged 65–69 was 4%, in those aged 70–74 it was 7%, in those aged 75–79 it was 9%, in those aged 80–84 it was 16%, and in those aged 85 and over it was 26%.

The population of the United Arab Emirates (UAE) enjoys a high level of health as measured by life expectancy (76.7 years), under-five mortality (7.2 per 1000 live births), and infant mortality (6.2 per 1000 live births) although levels of chronic disease risk factors are high and there is significant health loss from cardiovascular disease and diabetes (10). The age and sex structure of the UAE population is unusual as it is dominated by expatriate men and women of working age (11). This means that while the median age of the population is 33.3, only 2.3% are aged 60+ and 0.1% are aged 80+. However, rapid aging can be expected, so that by 2050, it is estimated that 23.5% of the population will be aged 60+ and 4.1% will be aged 80+.

Given the relationship between age and frailty and frailty and healthcare outcomes, these figures highlight the challenges that the UAE health and social care systems must address in the not-too-distant future. Population-based data on the scale of frailty amongst UAE elders are lacking. Also, the relationship between frailty and ethnicity, nationality, and culture has not been widely researched (12). Frailty may be more prevalent among UAE citizens as a result of the high burden of diabetes and cardiovascular disease (13). On the other hand, strong family networks may protect against the effects of frailty on health outcomes. Therefore, the objectives of this study were to determine the prevalence and correlates of frailty in a sample of community-dwelling older Emiratis.

## Materials and Methods

A cross-sectional study of Emirati adults aged 55 years and older was conducted in the emirate of Abu Dhabi, UAE, between September and December 2014. Obtaining probability samples for population research in UAE is challenging (14) for a number of reasons not least being the lack of suitable sampling frames, so in this study participants were recruited from among community-dwelling older people living in different areas of the UAE who visited the primary healthcare facilities at Zayed Military Hospital, Abu Dhabi. It was acknowledged that this would give higher estimates of frailty prevalence. The targeted sample size was 160, based on an assumed true frailty prevalence of 30% and an acceptable precision of ±7%. Persons aged 0–54, non-UAE nationals and non-Arabic speakers, were excluded. To minimize inter-rater variation, all interviews and measurements were performed by a single researcher (Saleha Jaber Al-Kuwaiti). This study was carried out in accordance with the recommendations of the Research Ethics Committee of Zayed Military Hospital. All participants received information and provided informed written consent in accordance with the Declaration of Helsinki.

### Data Collection

An interviewer-administered questionnaire, in Arabic, obtained information on age, gender, and marital status. Respondents were asked whether they lived alone and if they had experienced unintentional weight loss, exhaustion, or slow walking speed. Respondents were then asked about comorbidities, regular use of medication, recent hospitalization, use of vitamin D supplements, and how they rated their general health and overall memory.

### Measurements

Grip strength was measured in the right hand using an analog hand dynamometer with participants seated, elbow by their side and flexed to right angle, and a neutral wrist position. The dynamometer handle position was adjusted to fit the subject’s hand, and the average of three measurements of peak grip strength was recorded. A standard “timed up and go test (TUG)” was carried out. Subjects rose from an armchair and walked 3 m at normal pace, with usual footwear and walking aid as necessary, before returning to the chair and resuming the sitting position. The time in seconds taken to accomplish this exercise was recorded. Forced expiratory volume (FEV) was measured as a potential correlate of frailty using a standard clinic Vitalograph^©^ (15). Mental state was assessed using a modification of the mini-mental state examination (MMSE) (16). Some items were excluded from the MMSE test to make it more applicable to the subjects in this study, these included spelling, reading, and drawing. Subjects were assessed and scored for orientation, memory, attention, and calculation. The total possible score for the modified instrument was 26.

### Definitions

Five components made up the frailty phenotype used in this study. These are mostly retained from those proposed by Fried although changes were required to meet the particular needs of the study population. Unintentional weight loss was categorized as self-reported weight loss of 4.5 kg or more in the previous year. Self-reported exhaustion was defined as an affirmative response to the question “in the last month have you felt fatigue/exhaustion/tiredness or weakness.” Slow walking speed was also self-reported. Muscle weakness was defined as having hand-grip strength less than the lower 95% confidence interval for age group and gender taken from a table of normative data obtained in accordance with the American Society of Hand Therapists recommendations in multinational settings (17). Low physical activity was defined as having a TUG time greater than 12 s, which approximates the upper 95% confidence interval from normative data collected on a sample of community-dwelling elderly people with independent functioning (18). Frailty was defined as the presence of three or more frailty components while pre-frailty was defined as the presence of two or more components.

### Statistical Analysis

All data were normally distributed except mini-mental status examination, even after log transformation. In the univariate analysis, categorical variables are presented as frequencies and percentages and continuous variables are presented as means ± SD. The distribution of the characteristics of the study population for each frailty state is tabulated. For characteristics that are categorical variables, frequencies and percentages are shown and the significance of any differences in distribution is assessed by using chi-square and Fisher’s exact tests. For characteristics that are continuous variables, means are shown and one-way ANOVA tests are used. Univariate binary logistic regression analysis was performed to assess the association between frailty (outcome variable) and selected correlates (independent variables). Finally, the variables having *P*-values <0.2 were included in a multiple logistic regression model and a stepwise backward elimination method was adopted to adjust for confounders and identify the independent factors associated with frailty. Microsoft Excel 2010^©^ was used for data entry and analyses were conducted using Stata (version 13). Statistical significance was defined as *P*-values <0.05 and 95% confidence intervals.

## Results

The mean age of the study participants (*n* = 160) was 65 years and 56% (*n* = 90) were male. The characteristics of the study participants are summarized in Table 1. The majority of participants were married (83%) and few (15%) lived alone. Frailty characteristics were generally widespread among participants, with 20% reporting unintentional weight loss, 67% reporting exhaustion, and 68% reporting slow walking speed while mean grip strength was 42.7 kg and mean TUG time was 15.2 s. Most participants (85%) admitted to having comorbid conditions, 23% had been admitted to hospital at least once during the previous year, 40% were taking more than five medications, and 58% were taking vitamin D supplements. Eighty-one percent of participants described their health status as excellent or good, whereas 19% said it was poor. Similar replies were obtained when participants were asked about memory status. Two possible correlates of frailty were measured, mean FEV was 223 ml and the mean score on the modified MMSE was 21.5 (maximum possible score 26).

**Table 1:** **Characteristics of community-dwelling older Emirati citizens (*n* = 160^a^) attending primary healthcare services, Abu Dhabi, 2014**.

Variable	Descriptive statistics
**SOCIODEMOGRAPHIC CHARACTERISTICS**	
Age in years (mean ± SD)	65.6 ± 6.2
Age group, *n* (%)
55–59 years	22 (13.8)
60–64 years	54 (33.8)
65–69 years	41 (25.6)
70–74 years	25 (15.6)
≥75 years	18 (11.2)
Gender, *n* (%)
Male	90 (56.3)
Female	70 (43.7)
Marital status, *n* (%)
Married	133 (83.1)
Unmarried/divorced/widow(er)	27 (16.9)
Live alone, *n* (%)
Yes	24 (15.0)
No	136 (85.0)
**FRAILTY INDICATORS**	
Unintentional weight loss, *n* (%)
Yes	33 (20.6)
No	127 (79.4)
Self-reported exhaustion, *n* (%)
Yes	107 (67.0)
No	53 (33.0)
Slow walking speed, *n* (%)
Yes	109 (68.5)
No	50 (31.5)
Grip strength^b^ (kg, mean ± SD)	42.7 ± 14.5
Timed up and go test^c^ (s, mean ± SD)	15.2 ± 6.5
**FRAILTY CORRELATES**	
Comorbid conditions, *n* (%)
Yes	136 (85.5)
No	23 (14.5)
Use of more than five medications, *n* (%)
Yes	65 (40.6)
No	95 (59.4)
Number of admissions to hospital in past 1 year, *n* (%)
No admission	123 (76.9)
≥1 admissions	37 (23.1)
Use of vitamin D supplement, *n* (%)
Yes	94 (58.7)
No	66 (41.3)
Self-reported health status, *n* (%)
Excellent	7 (4.4)
Good	123 (76.9)
Poor	30 (18.8)
Self-reported memory status, *n* (%)
Excellent	17 (10.6)
Good	120 (75.0)
Poor	23 (14.4)
Forced expiratory volume (ml, mean ± SD)	223.6 ± 79.9
MMSE (score range, 0–26) (mean ± SD)	21.5 ± 4.2

*^a^Total do not always sum to n = 160 because of missing values*.

*^b^Grip strength cutoffs (kilograms) are as follows: age 55–59: male = 36.7, female = 26.4; age 60–64: male = 36.8, female = 22.2; age 65–69: male = 35.4, female = 22.5; age 70–74: male = 32, female = 20.7; age 75+: male = 12.7, female = 16.0*.

*^c^Timed up and go test cutoff = 12 s*.

Among this sample of community-dwelling older Emirati citizens attending primary healthcare services, frailty was common. The prevalence of frailty (95% CI) was 47% (39–55) and the prevalence of pre-frailty was 33% (26–41). Higher levels of frailty were seen in the older age groups, among women and among those who were unmarried, divorced, or widowed. Frailty prevalence did not vary by living status. Frailty was more common in those with comorbid conditions, those using more than five medications, those admitted to hospital within the previous year, and those reporting poor memory. The prevalence of frailty did not vary with vitamin D supplementation or self-reported health status. Frailty was associated with lower MMSE score and lower FEV. Table 2 summarizes these findings.

**Table 2:** **Frailty status according to the characteristics of the subjects and unadjusted odds ratio of frailty in community-dwelling older Emirati citizens (*n* = 160) attending primary healthcare services, Abu Dhabi, 2014**.

Variables	*N*	Frailty				Odds ratio^a^	95% CI	*P*-value^c^
		Frail	Pre-frail	Not frail	*P*-value^b^	
Overall frailty, *n* (%) (95% CI)	151	75 (46.9) (39.2–55.2)	53 (33.1) (25.9–40.9)	23 (14.4) (9.1–20.3)				
Age in years (mean ± SD)	160	68.3 ± 5.6	62.4 ± 4.4	60.6 ± 2.4	<0.001	1.31	1.20–1.43	<0.001
Age in categories, *n* (%)
55–59 years	22	3 (13.6)	12 (54.5)	7 (31.8)	<0.001			
60–64 years	53	17 (32.1)	22 (41.5)	14 (26.4)				
65–69 years	40	21 (52.5)	17 (42.5)	2 (5.0)				
70–74 years	24	23 (95.8)	1 (4.2)	0 (0.0)				
≥75 years	12	11 (91.7)	1 (8.3)	0 (0.0)				
Gender, *n* (%)
Male	86	31 (36.0)	37 (43.0)	18 (20.9)	<0.001	1		
Female	65	44 (67.7)	16 (24.6)	5 (7.7)		3.71	1.88–7.35	<0.001
Marital status, *n* (%)
Married	127	58 (45.7)	46 (36.2)	23 (18.1)	0.018	1		
Unmarried/divorced/widow(er)	24	17 (70.8)	7 (29.2)	0 (0.0)		2.89	1.12–7.45	0.028
Live alone, *n* (%)
Yes	23	12 (52.2)	10 (43.5)	1 (4.3)	0.294	1.13	0.46–2.74	0.794
No	128	63 (49.2)	43 (33.6)	22 (17.2)				
Comorbid conditions, *n* (%)
Yes	128	72 (56.3)	43 (33.6)	13 (10.2)	<0.001	1		
No	22	2 (9.1)	10 (45.5)	10 (45.5)		12.86	2.88–57.33	0.001
Use of more than five medications, *n* (%)
Yes	59	42 (71.2)	17 (28.8)	0 (0.0)	<0.001	1		
No	92	33 (35.9)	36 (39.1)	23 (25.0)		4.42	2.18–8.95	<0.001
Number of admissions to hospital in past 1 year, *n* (%)
≥1 Admissions	32	21 (65.6)	11 (34.4)	0 (0.0)	0.008	1		
No admission	119	54 (45.4)	42 (35.3)	23 (19.3)		2.30	1.02–5.19	0.045
Use of vitamin D supplement, *n* (%)
Yes	90	48 (53.3)	27 (30.0)	15 (16.7)	0.280	1.44	0.75–2.77	0.275
No	61	27 (44.3)	26 (42.6)	8 (13.1)		1		
Self-reported health status, *n* (%)
Excellent/good	122	56 (45.9)	45 (36.9)	21 (17.2)	0.161	0.45	0.19–1.03	0.061
Poor	29	19 (65.5)	8 (27.6)	2 (6.9)		1		
Self-reported memory status, *n* (%)
Excellent	17	6 (35.3)	5 (29.4)	6 (35.3)	0.022			
Good	114	55 (48.2)	42 (36.8)	17 (14.9)		1		
Poor	20	14 (70.0)	6 (30.0)	0 (0.0)		2.68	0.97–7.40	0.057
Forced expiratory volume (mean ± SD)	160	188.1 ± 49.1	262.1 ± 80.5	280.7 ± 87.1	<0.001	0.17	0.09–0.31	<0.001
MMSE score (mean ± SD)	160	19.7 ± 3.8	23.7 ± 2.6	24.7 ± 1.5	<0.001	0.64	0.56–0.74	<0.001

*n, frequency; CI, confidence interval*.

*^a^For the purpose of calculating the odds ratio, frailty was defined as the presence of three or more components of the frailty phenotype. For the continuous independent variables, the OR shown is the increase or decrease in odds of frailty per unit change in the variable as follows: age = 1 year, FEV = 1 SD, and MMSE = one point change in score*.

*^b^Chi-square test or Fisher’s exact test was used for categorical variables and one-way ANOVA was used for continuous variables*.

*^c^These P-values refer to univariate binary logistic regression analysis*.

The association between frailty as an outcome variable and selected population characteristics as independent variables is also summarized in Table 2. For the purpose of this analysis, only frailty (as defined by the presence of three or more frailty components) is used. The positive association of frailty with older age, female gender, non-married status, the presence of comorbid conditions, hospital admission, and use of more than five medications and the negative association of frailty with FEV and MMSE score are confirmed. After adjustment in the multivariate model, only age and gender retained their association with frailty (Table 3).

**Table 3:** **Multiple logistic regression analysis and adjusted odds of frailty and independent variables among community-dwelling older Emirati citizens (n = 160) attending primary healthcare services, Abu Dhabi, 2014**.

Variables	Frailty-adjusted odds ratio	95% CI	P-value
Age	1.28	1.11–1.46	<0.001
Gender
Male	1		
Female	3.23	1.02–10.24	0.046
Number of hospital admissions
No admission	1		
≥1 admissions	3.34	0.92–11.99	0.065
Comorbid condition
No	1		
Yes	4.40	0.66–28.57	0.122
Health status
Poor	1		
Good/excellent	0.31	0.08–1.30	0.110
Forced expiratory volume	0.45	0.22–1.00	0.052
Mini-mental status examination score	0.87	0.72–1.05	0.157

*n = frequency; CI = confidence interval*.

*Only associations with P < 0.2 are reported*.

## Discussion

We found a high prevalence (47%) of frailty in our sample of community-dwelling older Emirati citizens attending primary healthcare services. To the best of our knowledge, no population-based data are available from previous studies investigating frailty in the UAE. The prevalence of frailty in our study was higher than that seen in many other studies. However, comparison of frailty levels in our sample with those found in other populations and settings is problematic for two main reasons. First, although we used the five components of the FFI to define frailty, these can be operationalized in different ways, so that studies are not comparable even when they purport to use the FFI. Second, we studied a convenience sample of community-dwelling older Emirati citizens attending primary healthcare services rather than a random sample of the general elderly population. It is to be expected that our sample would have higher levels of frailty notwithstanding any environmental, ethnic, or cultural differences. That said, in a large representative sample of older people in the United States, among those in residential care the prevalence of frailty was 30% and among those with an overnight hospitalization in the previous 12 months it was 42% (19).

As reported in previous studies, we confirmed higher levels of frailty at older ages and among women (20). We did not find independent associations between frailty and comorbid conditions, hospital admission, and being non-married although such associations have been reported before. Also, we did not show associations with some other frailty correlates that have been reported in other studies, for example, cognitive function as measured by the MMSE (21) and FEV1. A high proportion of participants were taking vitamin D supplements which may not be unanticipated given that vitamin D deficiency is widely publicized in the UAE and has been shown to be associated with frailty in older men (22), but we did not show an independent association between frailty and supplementation or other medication use. Our study had modest power which may explain these failures also much of our data are based on self-reports and frail participants may have poor recollection that they have been started on vitamin supplementation.

We did not collect data on educational attainment, income, or ethnicity, factors that have been shown to be associated with frailty, because these would not have been relevant or appropriate questions for elderly Emiratis. Also we have not been able to comment on whether frail participants in our study experienced an increased risk of adverse outcomes or whether cultural and environmental factors may mitigate against such outcomes. These may be topics for future studies.

Our findings must be interpreted in light of the acknowledged limitations of this study. The non-random and selective nature of the sample limits generalizability to the wider general population of elderly Emiratis and prevents thorough comparison with other studies. The modest sample size widens the confidence intervals on the estimates of prevalence and reduces the power of the study to show small associations between frailty and potential explanatory variables. Our findings are based on cross-sectional data, and so the direction of relationships between frailty and potential correlates cannot be determined. Information on three of the five components of frailty was obtained by self-reports, and this carries the inevitable concern about the overall validity of the FFI as a measure of frailty and a predictor of health outcomes. Finally, as might be expected in an elderly population, there was correlation between some of the independent variables notably age and FEV and MMSE score. Although the fitted regression models did not show signs of multicollinearity, we cannot be certain that the estimated coefficients are unaffected.

## Conclusion

For the first time, using the five components of the FFI, we have reported a high prevalence of frailty among older Emiratis. Given that frailty is associated with adverse health outcomes and can be a means of identifying opportunities for intervention in clinical practice and health policy, the high prevalence that we have found merits further attention and consideration within professional and public health policy circles. We would propose a UAE action plan comprising a program of frailty research including longitudinal studies combined with the development of best practice guidelines for use in clinical and social care settings.

## Author Contributions

SA-K devised and designed the study and collected the data. All authors contributed to the analysis and interpretation of the data. The manuscript was drafted by SA-K and IB. All authors read and approved the final manuscript.

## Conflict of Interest Statement

The authors declare that the research was conducted in the absence of any commercial or financial relationships that could be construed as a potential conflict of interest.
